# Examining Sexual Well-being across the Lifespan: Assessing the Relationship between Sexual Satisfaction and Adjustment to Aging

**DOI:** 10.1192/j.eurpsy.2024.706

**Published:** 2024-08-27

**Authors:** I. Miguel, S. von Humboldt, I. Leal

**Affiliations:** ^1^Portucalense Institute of Psychology (I2P) & Department of Psychology and Education - Portucalense University, Porto; ^2^William James Center for Research, ISPA – Instituto Universitário, Lisbon, Portugal

## Abstract

**Introduction:**

Sexual satisfaction is relevant to aging well, throughout the lifespan.

**Objectives:**

This study aims to compare the perspectives of sexual satisfaction and adjustment to aging in three age cohorts, across the life span; and to analyze whether sexual satisfaction influences the perceptions of AtA.

**Methods:**

This cross-sectional study comprised participants from three different age cohorts (18-44; 45-64; and 65+ years). Four measures were used to meet the defined objectives: (a) Adjustment to Aging Scale (ATAS); (b) New Sexual Satisfaction Scale (NISS-S); and (c) sociodemographic, health and lifestyle questionnaire. Data were subject to One-way ANOVAs and hierarchical regression analyses.

**Results:**

Social support emerged as the most relevant dimension in the multifactorial nature of AtA. Generational differences were found in sense of purpose and ambitions [F (2, 616) = 14.203, p = .000], social support [F (2, 616) = 10.65, p = .000] and body and health [F (2, 616) = 8.73, p = .000]. Participants aged 65 and older showed significantly lower levels of sense of purpose and ambition, body and health, and social support. Younger participants showed the highest score for body and health. Age-related decreases in sexual satisfaction were also found, as younger participants showed statistically higher levels of sexual satisfaction, followed by middle-aged and older participants. Sexual satisfaction predicts all the dimensions of AtA, exception made for aging in place and stability, where age is the main predictor. Ego-centered sexual satisfaction positively predicted sense of purpose and ambitions (β = .212, p < .05) and social support (β = .311, p < .001); while partner/sexual activity centered sexual satisfaction was a positive predictor of zest and spirituality (β = .255, p < .01), body and health (β = .239, p < .001), and social support (β = .168, p < .05).

**Conclusions:**

Sexual satisfaction decreases with age and is positively related to all dimensions of AtA, hence gerontological interventions and program policies with older people would strongly benefit of including sexual satisfaction as a relevant variable for aging well.

Keywords: sexual well-being; sexual satisfaction; adjustment to aging; generational groups; lifespan.

**Disclosure of Interest:**

None Declared

